# Percutaneous Coronary Intervention and vascular access complications: A contemporary review

**DOI:** 10.1038/s44325-026-00125-6

**Published:** 2026-04-28

**Authors:** Tanawat Attachaipanich, Muzamil Khawaja, Edwin A. Takahashi, Hafeez Ul Hassan Virk, Mahboob Alam, Ian C. Gilchrist, Chayakrit Krittanawong

**Affiliations:** 1 Department of Internal Medicine, University of Missouri-Kansas City School of Medicine, Kansas City, MO USA; 2 Department of Cardiology, Emory University, Atlanta, GA USA; 3 Department of Radiology, Division of Vascular and Interventional Radiology, Mayo Clinic, Rochester, MN USA; 4 Division of Cardiovascular Disease, Department of Medicine, Albert Einstein Healthcare Network, Philadelphia, PA USA; 5 Texas Heart Institute and Baylor College of Medicine, Houston, TX USA; 6 Geisel School of Medicine at Dartmouth, Section of Cardiovascular Medicine, Heart and Vascular Center, Dartmouth Hitchcock Medical Center, Hanover, NH USA; 7 Penn Highlands Healthcare, Philadelphia, PA USA; 8 HumanX, Delaware, DE USA

**Keywords:** Cardiology, Diseases, Medical research, Risk factors

## Abstract

Transradial approach (TRA) is preferred over transfemoral approach (TFA) for percutaneous coronary interventions due to lower bleeding, vascular complications, and mortality, particularly in acute coronary syndrome. Radial access complications included intraprocedural events (spasm, dissection, and perforation) and postprocedural events (radial artery occlusion, hematoma, pseudoaneurysm, arteriovenous fistula, nerve injury, and infection). TFA remains necessary in certain settings but carries higher complication risk. Optimizing patient selection and procedural planning is essential to minimize vascular complications.

## Introduction

The transradial approach (TRA) has become the preferred access site for coronary angiography (CAG) and percutaneous coronary intervention (PCI)^1,2^. Evidence from large randomized controlled trials and meta-analyses shows that TRA reduces the risk of major bleeding and mortality in patients with acute coronary syndrome (ACS), particularly in high-risk populations^3–5^. Multiple large randomized studies have also demonstrated that TRA is associated with a lower risk of vascular complications compared with the traditional transfemoral approach (TFA)^4–8^. Reported vascular complication rates for TRA range from 0.1% to 1.4%, while those for TFA range from 0.4% to 3.7%^4,6,9^. Importantly, clinical presentation significantly influences both the incidence and clinical impact of access-site complications. Data from meta-analysis demonstrate that patients with ACS have higher complication rates compared with those with stable coronary artery disease (CAD). Major bleeding increases progressively across presentations, from stable CAD (TRA 0.4% vs. TFA 2.1%) to NSTE-ACS (0.9% vs. 1.3%) and STEMI (1.5% vs. 2.9%)^10^. Similarly, major vascular complications with TRA are higher in ACS than stable CAD (0.3% vs. 0.2%)^10^. These findings underscore that both baseline risk and the absolute benefit of access-site selection vary according to clinical context.

In ACS, particularly STEMI, TRA provides clinical benefits beyond reduction in access-site complications. In the RIVAL trial (*n* = 7021), TRA significantly reduced major vascular complications, with improved outcomes observed in high-risk subgroups^6^. The MATRIX Access trial (*n* = 8404) further demonstrated that TRA reduced the primary composite outcome, driven by lower major bleeding and all-cause mortality, with benefits largely attributable to reductions in access-site bleeding^4^. Consistently, a meta-analysis of 24 studies (*n* = 22,843) confirmed that TRA reduces mortality, ischemic events, major bleeding, and vascular complications across ACS presentations^10^.

**Fig. 1:**
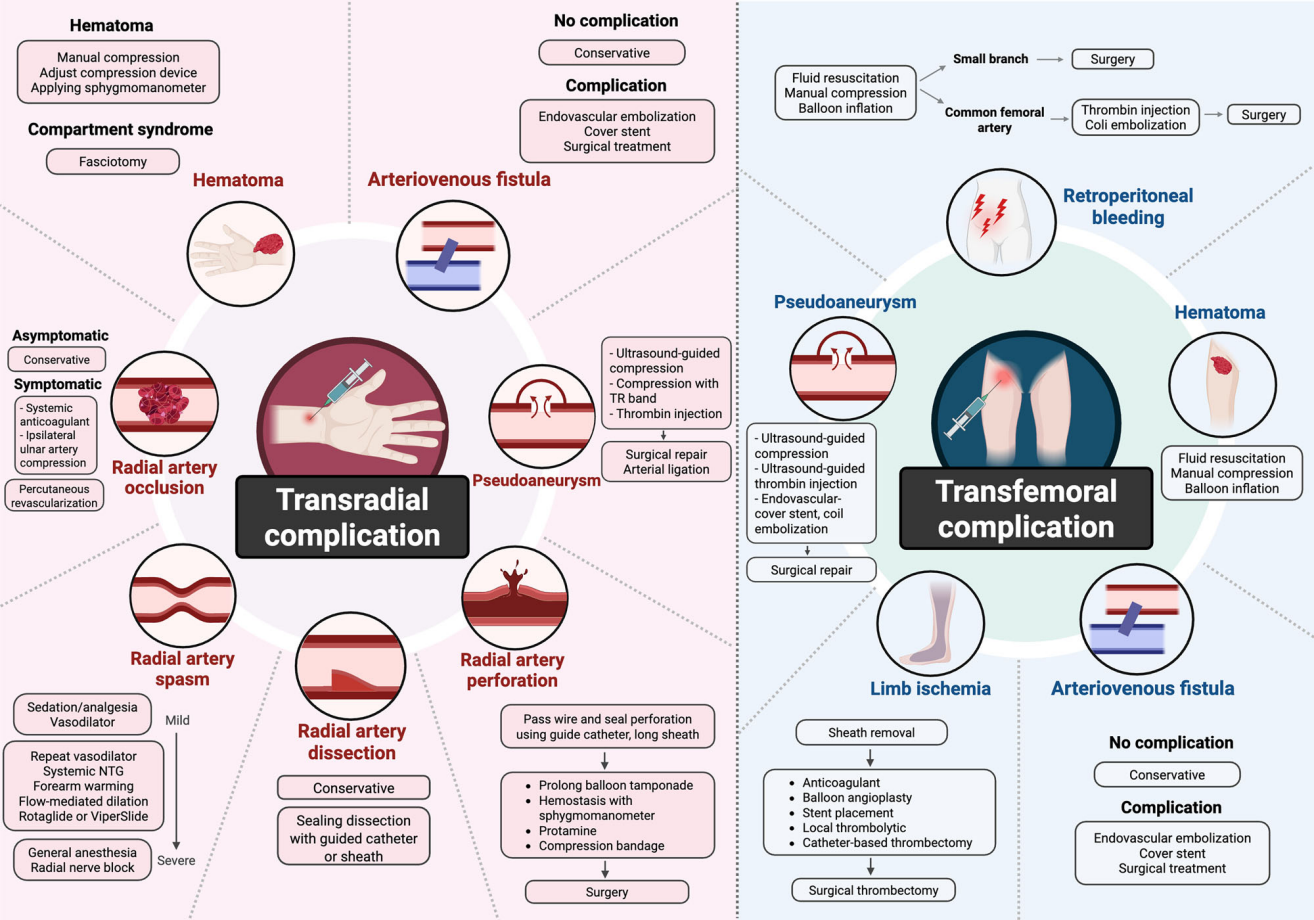
Vascular access-related complications and management strategies for transradial and transfemoral approaches. Schematic overview of vascular access–related complications associated with transradial (left) and transfemoral (right) approaches during coronary angiography and percutaneous coronary intervention. Transradial complications include radial artery occlusion, spasm, dissection, perforation, hematoma, pseudoaneurysm, and arteriovenous fistula, which are generally managed conservatively or with targeted endovascular interventions. Transfemoral complications include hematoma, pseudoaneurysm, retroperitoneal bleeding, limb ischemia, and arteriovenous fistula, often requiring more intensive management, including endovascular or surgical treatment. Created in BioRender. Attachaipanich, T. (2026) https://BioRender.com/0ujc9bi

In contrast, in stable CAD, the benefits of TRA are primarily limited to reductions in bleeding and vascular complications, without a consistent effect on mortality or major adverse cardiovascular events (MACEs). Subgroup analyses from randomized trials and meta-analyses support lower complication rates with TRA in stable CAD, although high-quality dedicated randomized data remain limited^10^. Trials such as OCTOPLUS (*n* = 377) and SAFE-PCI for Women (*n* = 1787) further demonstrate reductions in vascular and bleeding complications in predominantly stable populations^7,11^. Collectively, these data highlight that while TRA consistently reduces access-site complications across clinical settings, its clinical impact is greatest in ACS, where higher baseline risk amplifies the benefits of bleeding avoidance and translates into improved survival. Clinical outcomes comparing TRA and TFA from major randomized trials in patients with ACS and stable CAD are summarized in Tables 1 and 2, respectively. This review summarizes the contemporary evidence on vascular access complications in PCI, highlighting their etiology, management, prevention, and future directions, with an overview of key complications and management strategies illustrated in Fig. 1.

**Table 1:** Summary of major randomized clinical trials comparing transradial and transfemoral access in ACS patients

Study	Population	TRA vs TFA	Mortality	Major bleeding	Vascular complication	Ref
RIVAL (2012)	ACS	TRA 3507 vs TFA 3514	1.3% TRA vs 1.5% TFA; HR 0.86 (95%CI 0.58–1.29); *p* = 0.47	Non-CABG major bleeding 0.7% TRA vs 0.9% TFA; HR 0.73 (95%CI 0.43–1.23); *p* = 0.23	Major vascular complication: 1.4% TRA vs 3.7% TFA; HR 0.37 (95%CI 0.27–0.52); *p* = <0.0001 Large hematoma: 1.2% TRA vs 3% TFA; HR 0.40 (95%CI 0.28–0.57); *p* = <0.0001 Pseudoaneurysms needing closure: 0.2% TRA vs 0.6% TFA; HR 0.30 (95%CI 0.13–0.71); *p* = 0.006 Arteriovenous fistula: 0% TRA vs 0.1% TFA Ischemic limb needed surgery: 0% TRA vs 0% TFA	^6^
RIFLE-STEACS (2012)	STEMI	TRA 500 vs TFA 501	5.2% TRA vs 9.2% TFA; *p* = 0.020	Non-CABG bleeding: 7.8% TRA vs 12.2% TFA; *p* = 0.026	NA	^3^
STEMI-RADIAL (2014)	STEMI	TRA 348 vs TFA 359	2.3% TRA vs 3.1% TFA; *p* = 0.64	Major bleeding 1.4% TRA vs 7.2% TFA; *p* = 0.0001	Vascular access complication: 0.3% TRA vs 0.8% TFA; *p* = 0.62 Hematoma >15 cm: 0.6% TRA vs 5.3% TFA; *p* = 0.0002	^9^
SAFE-PCI for Women (All randomized, 2014)	Women undergoing urgent or elective PCI or diagnostic CAG	TRA 893 (53.2% ACS) vs TFA 894 (56.5% ACS)	NA	BARC 2, 3, or 5 bleeding: 0.6% TRA vs 1.6% TFA	Pseudoaneurysm: 0.1% TRA vs 0.1% TFA Arterial occlusion 0% TRA vs 0% TFA Arteriovenous fistula 0% TRA vs 0% TFA	^11^
SAFE-PCI for Women (PCI subgroup, 2014)	Women undergoing urgent or elective PCI	TRA 345 (71.6% ACS) vs TFA 346 (72.8% ACS)	NA	BARC 2, 3, or 5 bleeding: 1.2% radial vs 2.9% femoral	Pseudoaneurysm: 0.3% TRA vs 0% TFA Arterial occlusion 0% TRA vs 0% TFA Arteriovenous fistula 0% TRA vs 0% TFA	^11^
MATRIX (2017)	MI (STEMI/ NSTEMI)	TRA 4197 vs TFA 4207	1.6% TRA vs 2.2% TFA; RR 0.72 (95%CI 0.53–0.99); *p* = 0.045	BARC 3 or 5: 1.6% TRA vs 2.3% TFA; RR 0.67 (95%CI 0.49–0.92); *p* = 0.0128	Surgical access site repair: 0.1% TRA vs 0.4% TFA; RR 0.27 (95%CI 0.09-0.80); *p* = 0.0115	^4^
SAFARI-STEMI (2019)	STEMI	TRA 1136 vs TFA 1156	1.5% TRA vs 1.3% TFA; HR 1.15 (95% CI 0.58–2.30); *p* = 0.69	TIMI non-CABG major or minor: 1.4% TRA vs 2.0% TFA; HR 0.71 (95%CI 0.38–1.33), *p* = 0.28	Retroperitoneal bleeding: 0% TRA vs 0.4% TFA; *p* = 0.06	^178^

*ACS* Acute coronary syndrome, *BARC* Bleeding Academic Research Consortium, *CABG* coronary artery bypass grafting, *CAG* coronary angiogram, *HR* hazard ratio, *MI* myocardial infarction, *NA* no information, *NSTEMI* non-ST-segment elevation myocardial infarction, *PCI* percutaneous coronary intervention, *RR* risk ratio*, STEMI* ST-segment elevation myocardial infarction, *TFA* transfemoral access*, TRA* transradial access.

**Table 2:** Summary of major randomized clinical trials comparing transradial and transfemoral access in stable CAD patients

Study	Population	TRA vs TFA	Mortality	Major bleeding	Vascular complication	Ref
ACCESS (1997)	Stable and unstable angina	TRA 300 vs TFA 300 (NA% stable CAD)	0.3% TRA vs 0% TFA; *p* = 0.172	Major access-site bleeding (Hb loss ≥2 mmol/L, transfusion or vascular repair): 0% TRA vs 2.0% TFA	0% TRA vs 1.0% TFA	^172^
OCTOPLUS (2004)	Patients underwent PCI or CAG.	TRA 192 vs TFA 185 (NA% stable CAD)	4.3% TRA vs 3.3% TFA	Composite vascular endpoint (vascular/ bleeding complications): 1.6% TRA vs 6.5% TFA; *p* = 0.029)	Large hematoma: 1.6% TRA vs 6.5% TFA; *p* = 0.03 Hematoma: 3.5% TRA vs 11.4% TFA; *p* = 0.003 Pseudoaneurysm: 0.5% TRA vs 1.1% TFA Stroke: 0% TRA vs 0.6% TFA	^7^
SAFE-PCI for Women (All randomized, 2014)	Women undergoing urgent or elective PCI or diagnostic CAG	TRA 893 (46.8% Non-ACS) vs TFA 894 (43.5% Non-ACS)	NA	BARC 2, 3, or 5 bleeding: 0.6% radial vs 1.6% femoral	Pseudoaneurysm: 0.1% TRA vs 0.1% TFA Arterial occlusion 0% TRA vs 0% TFA Arteriovenous fistula 0% TRA vs 0% TFA	^11^
SAFE-PCI for Women (PCI subgroup, 2014)	Women undergoing urgent or elective PCI	TRA 345 (28.4% Non-ACS) vs TFA 346 (27.2% Non-ACS)	NA	BARC 2, 3, or 5 bleeding: 1.2% radial vs 2.9% femoral	Pseudoaneurysm: 0.3% TRA vs 0% TFA Arterial occlusion 0% TRA vs 0% TFA Arteriovenous fistula 0% TRA vs 0% TFA	^11^
Hamilton (2025)	Stable CAD	TRA 3784 vs TFA 2374	0.8% TRA vs 1.5% TFA; *p* = 0.021	Major bleeding: 0.4% TRA vs 0.8% TFA; *p* = 0.039	Vessel dissection: 0.2% TRA vs 0.2% TFA; *p* = 0.64 Pseudoaneurysm: 0.1% TRA vs 0.7% TFA; *p* < 0.001 Retroperitoneal bleeding: 0% TRA vs 0.2% TFA; *p* = 0.031 Access site bleeding: 0% TRA vs 0.1% TFA Stroke: 0.1% TRA vs 0.3% TFA; *p* = 0.16	^8^

*ACS* acute coronary syndrome, *BARC* Bleeding Academic Research Consortium, *CAD* coronary artery disease, *CAG* coronary angiogram, *Hb* hemoglobin, *NA* no information, *PCI* percutaneous coronary intervention, *TFA* transfemoral access, *TRA* transradial access.

## Vascular complications common to both access sites

### Access-site bleeding and hematoma

Access-site bleeding and hematoma are common vascular complications of PCI for both TRA and TFA. Compared with TFA, TRA is associated with a lower rate of access-site hematoma^6,9,12,13^. Previous studies have reported the incidence of access-site hematoma after TRA to range from 1.2% to 2.6%^6,9,12,13^. However, one study reported that the incidence increased to 23% when routine ultrasound surveillance was performed, although the majority of cases were clinically insignificant^14^. Hematoma is also the most common periprocedural complication of TFA, with a reported incidence ranging from 2% to 12%^15^.

Access-site hematoma following TRA usually occurs due to inadequate hemostasis, such as improper positioning or insufficient inflation of the compression device. Reported risk factors include multiple arterial puncture attempts, radial artery perforation, inadequate compression with a radial compression device, and concomitant therapy with glycoprotein IIb/IIIa inhibitors (GPI)^16–18^. Risk factors for access-site hematoma following TFA include patient-related factors such as advanced age, female sex, low body mass index (BMI), chronic kidney disease (CKD), stroke, peripheral artery disease (PAD), and chronic pulmonary disease^15^. Procedural factors include thrombolytic therapy, use of GPI, and periprocedural heparin administration^15^.

The EASY classification has been proposed to grade radial access hematomas by size and extent: grade I (≤5 cm, superficial), grade II (≤10 cm with muscular infiltration), grade III (>10 cm extending below the elbow), grade IV (extending above the elbow), and grade V (associated with ischemic complications such as compartment syndrome)^19^. Due to the superficial location of the radial artery over the radius, conservative management is usually sufficient. This includes manual compression, repositioning or reinforcement of the compression device, and close monitoring^17,20^. Prevention strategies include ultrasound-guided puncture to minimize multiple attempts, appropriate anticoagulation, and ensuring proper placement and adequate inflation of the hemostasis device.

**Fig. 2:**
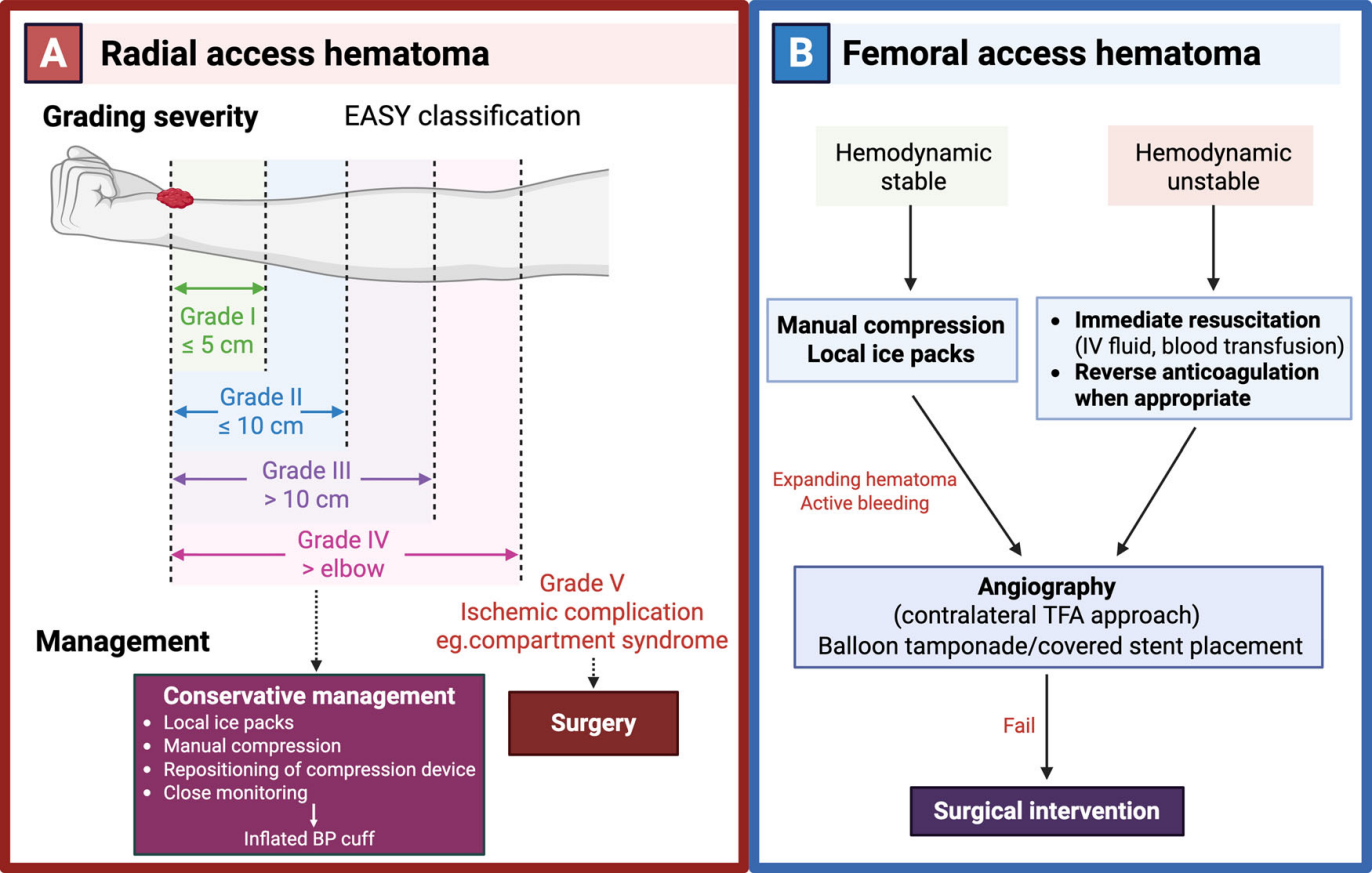
Management of access-site hematoma in transradial and transfemoral approaches. **A** Radial access hematoma classified according to the EASY grading system (Grades I-V) based on hematoma size and extent. Most cases (Grades I-IV) are managed conservatively with local measures, including manual compression, repositioning of the compression device, ice application, and close monitoring, with adjunctive use of an inflated blood pressure cuff when needed. Severe complications (Grade V), including ischemic injury or compartment syndrome, require urgent surgical intervention. **B** Femoral access hematoma management is guided by hemodynamic status. In stable patients, initial management includes manual compression and local measures. In unstable patients or those with ongoing bleeding, prompt resuscitation and reversal of anticoagulation are required. Expanding hematoma or active bleeding warrants angiographic evaluation, with endovascular treatment such as balloon tamponade or covered stent placement. Surgical intervention is reserved for cases refractory to endovascular therapy. Created in BioRender. Attachaipanich, T. (2026) https://BioRender.com/97g5rj4

However, femoral bleeding can progress rapidly and become life-threatening; therefore, early recognition and prompt management are critical. Superficial groin bleeding or small hematomas can usually be managed with manual compression, along with adjunctive measures such as local ice packs to reduce swelling and limit hematoma expansion^21^. In cases of hypotension, fluid resuscitation should be initiated promptly, and anticoagulation reversed when appropriate. If the diagnosis is uncertain or ultrasound findings are inconclusive, CT angiography can be considered as a noninvasive modality to confirm the presence and extent of bleeding and to identify active contrast extravasation. If manual compression fails to control bleeding, a sheath can be inserted via contralateral TFA, angiography performed to localize the bleeding site, and then balloon inflation used to achieve hemostasis^21^. A large multicenter randomized study comparing real-time ultrasound guidance with fluoroscopic guidance for femoral access demonstrated that ultrasound-guided vascular access was significantly associated with a lower incidence of large hematomas (≥5 cm) compared with fluoroscopic guidance^22^. Routine antibiotic prophylaxis is not indicated for femoral access-site hematomas following PCI, as these are typically sterile with a low risk of superinfection; antibiotic therapy should be reserved for cases with clinical evidence of infection^23^. An overview of hematoma classification and management strategies for TRA and TFA is shown in Fig. 2.

### Arterial pseudoaneurysm

Arterial pseudoaneurysm is a rare complication of PCI. It occurs when a penetrating arterial injury leads to hemorrhage and hematoma formation that communicates with the arterial lumen and is contained by the surrounding perivascular tissue^17^. The incidence of radial artery pseudoaneurysm has been reported ranging from 0.02% to 0.09% following TRA^6,17,24^. While the incidence of femoral artery pseudoaneurysm after TFA ranges from 0.05% to 8%^25–27^.

Several risk factors have been identified for pseudoaneurysm formation following TRA. Patient-related factors include advanced age, while procedural factors include multiple puncture attempts, excessive anticoagulation, large sheath size, inadequate compression after sheath removal, and catheter-related infection^18,28^. Similarly, risk factors for pseudoaneurysm following TFA include both patient- and procedural-related factors. Patient-related factors include advanced age, female sex, high BMI, low platelet count, hypertension, CKD, and PAD^29–32^. Procedural factors include prolonged or urgent procedures, use of large sheath size, puncture below the common femoral artery, use of antiplatelet or anticoagulant therapy, complex interventions, and inadequate hemostasis^29–32^. Puncture into the superficial or deep femoral artery or the external iliac artery also increases the risk of pseudoaneurysm formation^33^.

Management options for arterial pseudoaneurysm following TRA include ultrasound-guided compression, compression using a TR Band, or thrombin injection^12,17,34^. Surgical repair or arterial ligation can be considered if conservative management fails^17,28^. Prevention strategies focus on using ultrasound-guided access to minimize the number of puncture attempts, selecting appropriately sized sheaths, and ensuring adequate hemostasis after sheath removal.

For pseudoaneurysm following TFA, management options are broader and include observation, ultrasound-guided compression, ultrasound-guided thrombin injection, open surgical repair, and endovascular intervention^32^. Observation can be considered for small (<2 cm), stable pseudoaneurysms in patients not receiving anticoagulation, with serial ultrasound monitoring; spontaneous closure has been reported at a mean of 23 days^32,35–38^. Predictors of conservative management failure that require intervention include large size (>2 cm), narrow neck (<4 mm), enlarging sac, significant pain, early presentation (≤7 days after access), or ongoing anticoagulation^32,35^.

Ultrasound-guided compression involves applying probe pressure at the pseudoaneurysm neck in 10-minute cycles and has a reported success rate of 63–88%^32,36,37^. However, it is often painful requiring analgesia, time-consuming, and associated with recurrence rates up to 30%, increasing to 41–89% in anticoagulated patients^35,36^. Ultrasound-guided thrombin injection is now preferred over serial compression because of its higher success rate, reported at 93–100%^32,36,37^. This technique is faster, effective for noncompressible sites, and remains successful even in anticoagulated patients. The procedure is performed under local anesthesia by injecting thrombin directly into the pseudoaneurysm cavity under Doppler ultrasound guidance until complete thrombosis is achieved. Complications are uncommon; distal embolization occurs in 0.5–0.8% of cases, typically when the pseudoaneurysm neck is short and wide (>1 cm). Venous thrombosis occurs in approximately 0.2% of cases, with a higher risk in the presence of a concomitant arteriovenous fistula (AVF)^31,32,36,37^.

Surgical repair should be considered for rapidly expanding, infected, or complicated pseudoaneurysms, such as those causing compressive symptoms or ischemia, or when anatomic features increase the risk of complications or failure of thrombin injection, including wide-neck pseudoaneurysms and concomitant AVF^32,35^. Endovascular treatment, including covered stent placement or coil embolization, can be an alternative for patients with extensive comorbidities or high surgical risk^35,39^.

### Arteriovenous fistulas

AVFs are rare complications following PCI for both access sites. AVFs occur when both the arterial and venous walls are penetrated by the sheath or needle during the procedure^12,17^. The incidence of AVFs following TRA has been reported ranging from 0.1% to 0.2%^6,12,17,24,40^. While the incidence of AVFs after TFA ranges from 0.01% to 0.9%^41,42^.

The major risk factor for AVF formation following TRA is multiple puncture attempts, as the radial artery is surrounded by small superficial veins that may be inadvertently entered during access^18,43^. Several risk factors have also been identified for AVF formation following TFA. Patient-related factors include female sex and hypertension^41^. Procedural factors associated with increased risk include high-dose heparin use, use of warfarin, left groin puncture, and a low puncture site below the femoral bifurcation^41,44^. The higher risk associated with low puncture sites may be explained by the anatomy in the superficial and deep femoral arteries where the corresponding veins lie directly beneath the arteries, making simultaneous arterial and venous injury more likely, whereas the common femoral artery lies side by side with the vein, reducing this risk^44^. However, AVFs have also been reported above the bifurcation^44^.

Management of AVF following TRA usually conservative; observation or prolonged compressive bandaging is appropriate in most cases^12,17^. However, if an AVF leads to symptomatic arterial steal syndrome, venous congestion, or high-output cardiac physiology, intervention may be required. Options include endovascular embolization, covered stent placement, or surgical treatment such as ligation, excision, or vessel reconstruction^12,17,45^. Use of ultrasound-guided access can reduce the risk of AVF formation by lowering the number of vascular access attempts^40^.

Management of AVF following TFA is generally similar to that of radial AVF and depends on symptoms and persistence. Many AVFs close spontaneously; previous studies have shown that approximately one-third resolve within one year, with the majority closing in the first 4 months^41,46^. Importantly, previous study reported that persistent AVFs may remain stable without causing complications^41^. In cases of complicated or persistent AVFs, intervention may be considered, including ultrasound-guided compression, covered stent placement, or surgical repair^41,46^.

## Site-specific vascular complications

### Radial-specific complication

#### Radial artery spasm (RAS)

RAS is one of the most common intraprocedural radial complications, with a reported incidence of approximately 15%^47^. RAS is characterized by sudden, temporary narrowing of the radial artery, resulting in difficulty advancing the sheath or manipulating the catheter, often accompanied by patient pain^17,48^. The underlying mechanism of RAS relates to the high density of α-adrenergic receptors in the radial artery, which can be stimulated by sympathetic activity, local trauma, and catheter manipulation, leading to smooth muscle contraction^17^. Recent comprehensive reviews have summarized its incidence, risk factors, and management strategies^49^. Previous studies have reported that RAS accounts for up to one-third of transradial procedural failures^50^. RAS is associated with access-related complications including catheter entrapment, patient discomfort, prolonged procedural time, increased contrast exposure, and, importantly, the need for access site conversion^17,50,51^.

Several patient and procedural factors have been identified as predictors of RAS. Patient factors include younger age, female sex, BMI, smoking, diabetes, dyslipidemia, hypertension, PAD, and prior CABG^17,18,43,52,53^. Radial artery anatomy, including small vessel diameter and anatomical variants such as high take-off, is also associated with increased risk. Procedural risk factors include multiple puncture attempts, large sheath-to-artery ratios, higher contrast volume, and inadequate analgesia or sedation^17,18,43,52^. A novel biomarker, serum asymmetric dimethylarginine (ADMA), a natural analogue of L-arginine and inhibitor of nitric oxide production, has also been proposed as a predictor of RAS, with higher ADMA levels associated with increased risk^54^.

Management of RAS varies according to severity. Mild RAS should be treated by increasing sedation and analgesia and considering the addition of spasmolytic therapy, while minimizing catheter manipulation, which can worsen spasm^17^. For moderate to severe RAS, in addition to sedation, analgesia, and spasmolytics used in mild RAS, several treatment options have been proposed, including flow-mediated dilatation techniques, warm compression, subcutaneous nitroglycerin, forearm heating, and the use of lubricating solutions such as Rotaglide or ViperSlide^17^. A small randomized study also reported favorable outcomes with pressure-mediated dilatation using an automated angiographic injection system compared with vasodilator therapy^55^. In severe, refractory cases, sympathectomy via regional nerve block or general anesthesia may be required^17,56^.

Given the impact of RAS on procedural failure and access site conversion, preventive strategies are essential. Administration of intra-arterial radial cocktails, typically verapamil with or without nitroglycerin in combination with heparin, is recommended to reduce RAS^17^. A meta-analysis showed that subcutaneous nitroglycerin significantly reduced the rate of RAS; however, other routes, including intra-arterial and topical, did not show benefit^57^. Importantly, nitroglycerin administration was associated with an increased incidence of hypotension and should be used with caution^57^. Another meta-analysis also demonstrated the efficacy of verapamil in reducing RAS^58^.

Preprocedural strategies targeting the radial artery prior to puncture have also been explored. Topical vasodilators, particularly nitroglycerin applied locally before arterial access, have been shown to increase radial artery diameter and facilitate vascular access without significant systemic hemodynamic effects. In a randomized study (*n* = 100), preprocedural topical nitroglycerin significantly reduced the incidence of RAS and improved first-attempt puncture success compared with placebo^59^.

Certain sheaths, including hydrophilic-coated sheaths, significantly lower spasm rates compared with uncoated sheaths^60^. Moreover, smaller sheath sizes are preferred to reduce the risk of RAS, although sheath length appears to have no impact^60^. A randomized study comparing long versus short and hydrophilic-coated versus uncoated introducer sheaths demonstrated significantly lower RAS rates with hydrophilic-coated sheaths compared with uncoated sheath; however, there was no difference in RAS between long and short sheaths^60^. Large sheath-to-artery ratios should also be avoided, as higher ratios increase the risk of RAS^60^. A randomized study comparing 5 Fr and 6 Fr guiding catheters found a numerically lower RAS rate with smaller catheters, although the difference was not statistically significant^61^. Another randomized study comparing 6.5 Fr sheathless guiding catheters with 6.0 Fr slender sheaths showed no significant difference in RAS, despite the sheathless catheter group having a significantly higher sheath-to-artery ratio >1, which has previously been associated with increased RAS risk^62^.

Adequate analgesia and sedation, local anesthesia at the puncture site, ultrasound-guided vascular access to minimize puncture attempts, and reducing catheter manipulation or exchanges are also recommended to prevent RAS^17^. A large randomized study showed that procedural sedation with analgesia significantly reduced the incidence of RAS and decreased access-site crossover compared with no sedation^52^. A prospective study found that preprocedural ultrasound significantly reduced RAS^63^. For intra-procedural ultrasound-guided TRA, a randomized study showed improved procedural success rates but no significant difference in RAS incidence^64^. In the randomized RAUST trial (*n* = 698), real-time ultrasound guidance significantly reduced time to access, increased first-pass success, and decreased the number of difficult punctures compared with palpation, without significant differences in RAS, patient-reported pain, or bleeding^65^. In the SURF randomized trial (*n* = 1388), ultrasound guidance did not reduce clinical outcomes compared with standard access; however, it significantly improved procedural efficiency, including shorter access time, fewer attempts, lower rates of difficult access, and higher first-pass success^66^. Regarding access site selection, a meta-analysis showed that distal TRA was associated with lower RAS compared with traditional proximal TRA; however, distal TRA required a higher rate of access-site crossover^67^.

#### Radial artery perforation

Radial artery perforation is rare, with a reported incidence ranging from 0.1% to 1%^68,69^. Although uncommon, it can lead to hematoma formation and, if unrecognized, may progress to compartment syndrome. Perforation most often results from passage of the wire into small branches or from the consequence of the “razor effect”, where the tip of the guide catheter avulses the vessel wall during advancement^70^. Risk factors for radial artery perforation include advanced age, short stature, female sex, over-anticoagulation, aggressive wire manipulation, use of hydrophilic wires, and tortuous arteries^28,68^.

Management is usually successful with conservative measures aimed at sealing the perforation, such as advancing the catheter or a long sheath proximal to the site of injury^17,68,71^. In cases where the lesion can be crossed with a coronary guidewire (e.g., 0.014-inch workhorse wire), advancement of a catheter over the wire may allow continuation of the procedure. Adjunctive techniques such as balloon-assisted tracking or a mother-in-child approach may facilitate catheter delivery across the injured segment. Importantly, the catheter itself may provide temporary tamponade at the site of perforation, helping to seal the defect when maintained in position. If the wire cannot be passed proximal to the perforation, other strategies include local compression with a sphygmomanometer or compression bandage, manual compression, administration of protamine, or antegrade balloon inflation from an alternative access site^17^.

Prevention focuses on careful fluoroscopic guidance and appropriate wire selection. Using J-tipped wires reduces the risk of entering side branches compared with straight-tipped hydrophilic wires, which are more likely to deviate into small branches^72^. A recent randomized trial comparing a 1.5-mm Baby J-tipped hydrophilic guidewire with a standard 3-mm J-tipped wire reported low perforation rates in both groups (1.8% with the standard wire versus 0% with the Baby J wire), without a statistically significant difference^72^. Balloon-assisted tracking, advancing the guide catheter with a partially protruded coronary balloon to create a conical, smooth, and tapered tip, can also help navigate tortuous vessels and avoid the razor effect^70^. Importantly, if resistance is encountered during wire advancement or the patient reports discomfort or pain, advancement of the catheter and wire should be stopped, and angiography should be performed promptly to assess position and rule out complications^17^.

#### Radial artery dissection

Radial artery dissection is a rare complication of TRA, with a reported incidence of approximately 0.4%^60^. It occurs when the vessel wall is injured during advancement of the wire or catheter and is usually diagnosed early in the procedure. Previous studies identified radial artery dissection as a cause of procedural failure in up to 10% of cases^50^. Several studies have reported that conservative management is often successful, similar to the approach for radial artery perforation, including sealing the dissection with a sheath or guide catheter^17,20^. Radial artery dissections are often clinically benign, as dissection flaps are typically oriented opposite to the direction of blood flow; thus, antegrade flow tends to compress the flap against the vessel wall rather than propagate the dissection, making progression to vessel occlusion uncommon in most cases. Prevention strategies are similar to those used for perforation and include careful fluoroscopic guidance to define arterial anatomy and catheter position, along with appropriate wire selection^17,20^.

#### Radial artery anomalies

Radial artery anomalies are associated with a higher risk of procedural failure, access-site crossover, and potential complications, including spasm and perforation^17,73^. Previous angiographic and autopsy studies have reported the incidence of radial artery anomalies to be approximately 14%^73,74^. The most common anomaly is high-bifurcation, reported in about 7% of cases; the majority of these vessels have a small caliber (<3 mm). However, high-bifurcation alone has not been shown to increase procedural failure^73^. A radial artery loop is present in about 2.3% of cases, usually involving the proximal radial artery just below the brachial bifurcation. The loop is often accompanied by a recurrent radial artery at its apex and is associated with a high procedural failure rate (up to 37%) and an increased risk of perforation and dissection^73^. Furthermore, crossing a loop can predispose to RAS, which may lead to procedural failure. Extreme radial artery tortuosity has been reported in about 2% of cases and is associated with a procedural failure rate of 23% and an increased risk of RAS^73^. Given the relatively high prevalence and potential impact of radial artery anomalies, some experts recommend routine radial angiography to assess anatomy before intervention and plan an optimal procedural approach.

#### Brachial artery injury, perforation, and dissection

Brachial artery perforation or dissection is a rare complication of TRA, with a reported incidence of about 0.04%^75^. Only a few case reports have described brachial artery perforation or dissection^75,76^. Perforation typically occurs in small-caliber arterial segments or in the presence of anatomic anomalies such as tortuosity or loops^75^. Most cases can be managed conservatively without abandoning the radial approach^75^. One reported strategy involves crossing the perforated segment with a soft 0.014-inch coronary wire and then gently advancing a downsized or alternative catheter using a corkscrew maneuver to complete the procedure^75^. Pull-back angiography at the end of the procedure is recommended to confirm sealing of the perforation; long sheaths or balloon tamponade can be used if needed^75^. Flow-limiting brachial artery dissection has been treated successfully with stent implantation in selected cases^76^. The use of balloon-assisted tracking has also been reported to help overcome anatomic difficulties, including catheter-induced brachial perforation and subclavian artery dissection^77^.

#### Subclavian artery injury, perforation, and dissection

Vascular complications from TRA are more common in peripheral arteries than in central arteries. Subclavian artery injury, perforation, or dissection is an extremely rare^78^. Central vascular complications, including iatrogenic aortic dissection, are estimated to occur in <0.1% of cases^79^. Only a few case reports describe subclavian artery dissection or perforation^78,80–82^. A retrospective study of iatrogenic aortic dissection following cardiac catheterization showed that most patients were managed conservatively when hemodynamically stable, with excellent long-term outcomes^79^. However, a small number of patients developed cardiogenic shock or died and required surgical intervention^79^. Reported management strategies for subclavian artery perforation and dissection include conservative treatment, coil embolization, or stent placement via TFA^78,80–82^. Most cases reported resistance during guidewire advancement, particularly in the presence of anatomic kinking^78,80–82^. Using a smaller, more flexible guidewire or switching early to an alternative access site can help avoid these complications.

#### Catheter kinks

Catheter kinking is uncommon but can lead to vascular complications. Kinking occurs due to excessive catheter torque during manipulation^83^. Risk factors include anatomical tortuosity from the radial to the subclavian level, radial artery anomalies, use of small-caliber catheters, and the presence of RAS, which increases resistance^83–85^.

Management of catheter kinks should follow a stepwise approach. The first step is to rotate the catheter in the opposite direction of the initial torque and advance a guidewire to help unfold the kink. If unsuccessful, options include fixing the catheter tip externally by applying compression with a sphygmomanometer to stabilize the distal tip, encasing the knot with a longer sheath, or untwisting using hydraulic pressure. In refractory cases, internal fixation using a snare introduced through TFA can be performed to untwist the catheter^83–85^.

Prevention of catheter kinking involves careful catheter handling and procedural planning. Operators should avoid over-rotation of the catheter (>180 degrees), perform coronary cannulation with a guidewire inside the catheter during manipulation, and monitor for early warning signs such as loss of torque, damping of pressure, or inability to aspirate^83–85^. Equipment selection is also important: consider catheters and guides with higher kink resistance, use longer or reinforced sheaths, and employ polymer-jacketed guidewires (e.g., Glidewire) to navigate tortuous anatomy^83–85^. Patient and procedural preparation includes adequate sedation and spasmolytics to prevent RAS, considering left TRA in cases of marked right-sided tortuosity (especially in elderly patients where it is more prevalent), and early conversion to TFA if severe tortuosity or resistance is encountered^83–85^.

#### Catheter entrapment

Catheter entrapment is a rare complication, usually occurring in the setting of refractory severe RAS. The reported incidence is approximately 0.8%^86^. Although uncommon, catheter entrapment can have serious consequences; forceful removal may result in arterial avulsion requiring surgical intervention^87^. A stepwise approach to managing catheter or sheath entrapment has been proposed, similar to the management of moderate to severe RAS^17,84^. Initial measures include pharmacologic therapy with intra-arterial vasodilators such as nitroglycerin and/or verapamil, along with increased sedation using opioids or benzodiazepines^17,84^. If these are ineffective, non-pharmacologic strategies can be employed, including warm compression, forearm heating, and flow-mediated vasodilation using a sphygmomanometer^17,84^. For persistent entrapment, deep sedation may be considered, and in refractory cases, invasive techniques such as regional nerve block or surgical endarterectomy may be required^17,56,84^.

#### Radial artery occlusion (RAO)

RAO is the most common postprocedural complication of TRA, with reported incidence rates ranging from <1% to as high as 33%^88^. More recent randomized studies have reported early RAO rates between 0.8% and 10%^89^. Although the majority of RAO cases remain asymptomatic, early detection is important to preserve radial artery patency for future cardiac catheterization or use as a conduit for CABG^17^. The pathophysiology of RAO depends on timing. Acute RAO results from endothelial injury during the procedure, local hypercoagulability, and reduced blood flow caused by compressive hemostasis^90^. In contrast, chronic RAO is primarily due to vascular remodeling, including intimal thickening secondary to prior injury^91^.

Several predictors of RAO have been identified. Patient-related factors include advanced age, female sex, diabetes, and low BMI^14,18,43,92^. Procedural factors include sheath-to-artery ratio >1, inadequate anticoagulation, multiple puncture attempts, and RAS^14,18,43,92,93^. Postprocedural factors include prolonged or occlusive hemostasis^92,94^. In particular, prolonged radial compression exceeding 4 h strongly predicts RAO, emphasizing the importance of patent hemostasis and minimizing compression time^94^.

Management of RAO depends on whether the patient is symptomatic and whether radial patency is needed^17^. Many cases resolve spontaneously over time; a meta-analysis found RAO incidence decreased from 7.7% at 24 h to 5.5% at 1 month after the procedure^88^. Treatment is typically reserved for symptomatic patients and may include systemic anticoagulation, ipsilateral ulnar artery compression to augment radial flow, or, in select cases, percutaneous revascularization^17,95^. A prospective study reported high rates of recanalization after 4 weeks of low-molecular-weight heparin (LMWH) in patients with symptomatic RAO^95^. Another randomized study demonstrated the effectiveness of 5000 units of unfractionated heparin (UFH) combined with ipsilateral ulnar artery compression^96^.

Preventive strategies for RAO involve both intraprocedural and postprocedural measures. During the procedure, minimizing multiple puncture attempts is important, as failed and repeated punctures are predictors of RAO. A randomized study comparing real-time ultrasound-guided radial artery cannulation with palpation alone demonstrated improved success rates of arterial access, although RAO rates were not reported^89,93^. The risk of RAO increases when the sheath-to-artery diameter ratio exceeds 1; thus, reducing sheath and catheter size and using preprocedural ultrasound to guide sheath selection may help lower RAO risk.

Access site selection can also influence RAO. A recent randomized study showed that distal TRA reduced forearm RAO compared with proximal TRA but was associated with higher crossover rates^97^. Sheath size is also a key determinant of RAO, with a meta-analysis of 18 studies demonstrating a clear size-dependent relationship, as RAO rates increased from 0% with 4 Fr to 19.5% with 7 Fr sheaths^88^. In addition, a higher sheath-to-artery ratio has been strongly associated with increased RAO risk, particularly when the sheath outer diameter exceeds the radial artery inner diameter (ratio >1)^93^. In the randomized RAP and BEAT trials (*n* = 1838), 6 Fr Glidesheath Slender failed to demonstrate noninferiority to a standard 5 Fr sheath with respect to radial artery occlusion, underscoring the importance of sheath outer diameter in the development of RAO^98^. Sheathless guiding catheters may also reduce RAO risk. In a randomized study comparing 6.5 Fr sheathless guiding catheters with 6.0 Fr slender sheaths, the sheathless group showed a numerically lower RAO risk, although the difference was not statistically significant, despite a higher sheath-to-artery ratio (>1) in the sheathless group^62^.

Adequate intraprocedural anticoagulation is another key strategy. Current guidelines recommend high-dose UFH to prevent RAO, as thrombus formation plays a central role in acute RAO. A randomized study comparing high-dose UFH (100 IU/kg) with low-dose UFH (50 IU/kg) demonstrated superior efficacy of high-dose UFH in reducing RAO^14^. Moreover, a meta-analysis of randomized studies supported these findings^14^.

In the postprocedural period, patent hemostasis is a key strategy to prevent RAO. In contrast to occlusive compression, which promotes thrombus formation and is a predictor of RAO, patent hemostasis is defined as maintaining antegrade radial blood flow during compression^89^. Randomized studies have demonstrated a significant reduction in both early and late RAO with patent hemostasis compared with occlusive techniques^99,100^. The PROPHET trial showed that patent hemostasis significantly reduced RAO rates at both early (<24 h; 5% vs. 12%) and late (30-day; 1.8% vs. 7%) follow-up compared with occlusive compression^99^. The addition of ipsilateral ulnar artery compression to patent hemostasis may further reduce RAO risk. The rationale is that compressing the ulnar artery augments radial blood flow through the palmar arch and promotes vasodilator release^96^. In the large PROPHET II trial, ipsilateral ulnar compression in addition to patent hemostasis further reduced early RAO from 4.3% to 1% at 24 h and late RAO at 30 days from 3% to 1%^99^. Ipsilateral ulnar compression also improved the success rate of achieving patent hemostasis^99^.

The type of compression strategy, including dedicated radial compression devices (e.g., TR Band) and conventional compressive dressings, may influence RAO; however, contemporary data suggest comparable efficacy when optimal technique is applied. Randomized and prospective studies have demonstrated similar rates of radial artery patency between device-based compression and traditional elastic dressings^101,102^. More recent randomized data further support comparable early RAO rates, despite shorter hemostasis time and improved patient comfort with conventional dressings^103^. In contrast, earlier studies reported higher RAO rates with conventional pressure dressings, likely reflecting prolonged and non-titratable compression^104^.

While shorter compression duration increases the risk of hematoma, prolonged compression increases the risk of RAO^105^. Contemporary practice typically involves approximately 60 min after diagnostic angiography and 120–180 min following PCI^12^. Shorter compression durations have been consistently associated with lower RAO. In a retrospective cohort of 400 PCI patients, a 2-hour TR Band protocol reduced RAO compared with 6 h without increasing bleeding^106^. The CRASOC series similarly demonstrated lower RAO with shorter, mild compression (1.5 vs. 4 h), although with higher bleeding and recompression rates^107^. A network meta-analysis of 10 randomized trials (>4900 patients) identified a 2-hour protocol as providing the best balance between reducing RAO and avoiding bleeding; durations <90 min increased bleeding, while longer durations offered no additional benefit^108^. In a randomized trial (*n* = 129), an accelerated deflation protocol initiated 20 min after sheath removal reduced hemostasis time (113 vs. 131 min) without increasing RAO or hematoma^109^. Overall, compression ≤120 min appears optimal to minimize RAO while maintaining acceptable bleeding risk, with accelerated protocols feasible in selected patients under close monitoring.

Pre-puncture subcutaneous nitrates and post-procedural, pre-hemostasis intra-arterial nitrates have been proposed to reduce radial artery occlusion (RAO), primarily through their vasodilatory effects. In a multicenter randomized trial (*n* = 1706), postprocedural intra-arterial nitroglycerin administered before hemostasis significantly reduced early RAO compared with control (8.3% vs. 11.7%)^110^. Similarly, in a randomized study (*n* = 188), subcutaneous nitroglycerin injection at the radial puncture site reduced early RAO (5.4% vs. 14.4%)^111^. In contrast, the PATENS randomized trial (*n* = 2040) demonstrated no significant reduction in RAO with intra-arterial nitroglycerin, whether administered at sheath insertion or before sheath removal (1.8% vs. 1.6%), and was associated with a higher incidence of hypotension (2.5% vs. 1.2%)^112^. Collectively, these findings suggest that while vasodilators may mitigate radial artery spasm, they do not consistently prevent RAO. The low RAO rate (2.4%) observed in contemporary practice underscores the predominant role of optimized procedural strategies over pharmacologic adjuncts alone in RAO prevention.

#### Hematoma at the forearm

Forearm hematoma can occur remote from the access site, most often due to perforation of small radial branches during wire advancement. Several factors have been identified as predictors of bleeding and vascular complications, including hematoma formation. Patient-related factors such as advanced age, female sex, and diabetes are associated with an increased risk of bleeding and vascular complications^113,114^. Procedural factors include inadequate hemostasis, inappropriate compression device positioning, a large sheath-to-radial artery size ratio, and radial artery anomalies, all of which increase vascular risk^28,68^. The use of straight-tipped hydrophilic wires, which are more likely to deviate into small branches and cause avulsion or perforation, is another important risk factor^72^.

Management of forearm hematoma typically includes repositioning the compression device, applying an additional compression device, performing manual compression, using an Ace bandage wrap, and applying a blood pressure cuff proximal to the site, inflated to systolic pressure and gradually released over 1–2 h^17^. Additional supportive measures include local ice packs to reduce swelling and the application of compressive or elastic dressings to limit hematoma expansion. Prompt recognition is essential; if diagnosis is delayed or treatment is inadequate, large forearm hematomas can extend proximally and, if unrecognized, may progress to compartment syndrome, requiring surgical intervention^17^.

#### Compartment syndrome

Compartment syndrome is an uncommon but serious complication of TRA, resulting from large hemorrhage or hematoma of the radial artery, which can compromise both radial and ulnar arterial blood flow and lead to ischemia. The reported incidence approximately 0.05%^6,115^. The risk factors for developing compartment syndrome are similar to those associated with radial artery bleeding or perforation, including inadequate hemostasis, inappropriated compression device positioning, use of GPI,and high or low BMI^18,20^. Management requires emergent fasciotomy to decompress the affected compartment^17,28,115^. Prevention focuses on adequate hemostasis, proper compression device (e.g., TR band device) positioning, nursing staff education on hematoma management and early recognition of bleeding, and prompt control of access-site complications to avoid progression to compartment syndrome^17,28^.

#### Nerve Injury

Nerve injury following TRA is rare, with a reported incidence of 0.16%^116^. Unlike the femoral artery, the radial artery at the typical puncture site is anatomically separated from major nerves^28^. However, perforation of the recurrent radial artery during the procedure has been associated with posterior interosseous nerve injury, which can lead to thumb drop^117^. In addition to direct nerve trauma, extrinsic compression from a local hematoma can also cause nerve injury^28^. Risk factors for nerve injury are similar to those for hematoma and include multiple puncture attempts^28^. Most reported cases resolve spontaneously and are treated conservatively^28,117^. For persistent symptoms, treatment options include nonsteroidal anti-inflammatory drugs (NSAIDs), local corticosteroid injections, physical therapy, or selective serotonin reuptake inhibitors (SSRIs)^43^. Prevention focuses on strategies to reduce hematoma formation and early recognition of nerve injury, especially if the patient reports pain or paresthesia during the procedure^43,117^.

#### Infection

Infection at the radial access site is rare, with an incidence of approximately 0.1% reported in neurointerventional procedures; however, data on the incidence in the setting of cardiac catheterization, which generally involves shorter procedural times, are limited^118^. Mycotic aneurysms following TRA have been described in case reports^119^. Catheter-related infection is also recognized as a risk factor for radial artery pseudoaneurysm^17^. Reported radial access site infections have been primarily associated with longer procedures in which catheters or sheaths remain in place for prolonged periods, rather than short-duration coronary interventions^120^.

### Femoral-specific complications

#### Retrograde iliac dissection

The true incidence of retrograde iliac dissection is difficult to determine because many cases are asymptomatic^121^. This complication can occur during retrograde wire advancement or sheath angiography, particularly in the presence of tortuous iliac arteries and atherosclerotic plaque^122^. Management is typically conservative, as antegrade blood flow often seals the dissection spontaneously and prevents further complications^122^. Most non-flow-limiting dissections can therefore be managed conservatively, and the procedure can usually be continued with caution. However, rewiring within the dissection plane should be avoided to prevent propagation. When the dissection is not recognized early and extends, leading to luminal obstruction and limb ischemia, endovascular treatment with angioplasty or stenting is recommended^121^.

#### Retroperitoneal bleeding

Retroperitoneal bleeding is an infrequent but serious complication of TFA, with a reported incidence of 0.025% to 0.9%^123–125^. Its prevalence has declined over time with the increasing use of TRA^124^. Retroperitoneal bleeding is associated with significantly higher 30-day mortality and in-hospital MACEs^124^. Several risk factors have been identified. Patient-related factors include female sex, low body weight or body surface area, hypercholesterolemia, CKD, chronic obstructive pulmonary disease, cardiogenic shock, and presentation with acute myocardial infarction^124–127^. Procedural factors include large sheath size, high femoral puncture site, use of GPI, emergency procedures, and periprocedural heparin administration^124–127^. Bleeding can arise from the access site, especially when the puncture is high, or from inadvertent micropuncture of small vessels such as the inferior epigastric artery^125,128^. A high femoral puncture, defined as sheath entry above the inguinal ligament into the external iliac artery, or above the middle third of the femoral head on fluoroscopy, or above the inferior epigastric artery, predisposes to retroperitoneal bleeding because manual compression is less effective for hemostasis^125^. However, even puncture below the inguinal ligament does not eliminate the risk, as bleeding can track into the retroperitoneal space^125^.

Diagnostic evaluation depends on hemodynamic status. In hemodynamically stable patients with suspected retroperitoneal hematoma, CT angiography is the preferred noninvasive modality for confirming the diagnosis. However, in unstable patients, CT imaging may delay life-saving intervention; therefore, prompt return to the catheterization laboratory for immediate angiography to identify the bleeding site should be performed.

Management involves prompt fluid resuscitation, blood transfusion, and reversal of anticoagulation when indicated^126^. Bleeding often ceases spontaneously due to the tamponade effect of accumulating retroperitoneal blood. When the puncture site is high, manual compression is preferred, as studies have shown higher retroperitoneal bleeding risk with vascular closure devices such as Angio-Seal compared with manual compression, possibly due to thicker fascial and muscle layers proximally that limit device effectiveness^127,129^. In unstable patients, despite resuscitation, percutaneous treatment should be performed via contralateral TFA to localize the bleeding site by angiography. Prolonged balloon inflation can tamponade bleeding, and covered stents such as Viabahn self-expanding or iCAST balloon-expandable stents may be deployed when appropriate^126^. For bleeding at the common femoral artery, surgical consultation should be considered, particularly because stent placement in this location carries a risk of stent fracture and may limit future access. For small branch vessel injury, treatment options include thrombin injection or coil embolization depending on vessel size. Bleeding from the external iliac artery is typically managed with covered stent placement^126^. If percutaneous methods fail, urgent surgical intervention is required^126^.

#### Limb ischemia

Limb ischemia after TFA is uncommon, with a reported incidence ranging from less than 1% to 2%^130,131^. Risk factors include patient-related factors such as advanced age, PAD, and hypercoagulable states, as well as procedural factors such as a large sheath-to-artery ratio^131^. Ischemia may result from obstruction of blood flow by the sheath, particularly in patients with small femoral arteries or pre-existing PAD with impaired perfusion. If limb ischemia develops while a sheath remains in place, the first step in management is prompt sheath removal. Other mechanisms of limb ischemia include thrombosis, vessel dissection, or distal embolization^131,132^. Treatment options include systemic heparinization, percutaneous balloon angioplasty, stent placement, local infusion of thrombolytic agents, or catheter-based thrombectomy^131^. If percutaneous therapy fails, urgent vascular surgery consultation for surgical thrombectomy or exploration is indicated.

Limb ischemia may also occur after the use of vascular closure devices, with mechanisms varying by device type. For plug-based devices (e.g., Angio-Seal), ischemia can result from inadvertent collagen plug deployment inside the arterial lumen, malposition of the footplate in a small vessel or at a bifurcation, or footplate entrapment against posterior wall calcified plaque, causing occlusion^132–134^. Management typically involves percutaneous endovascular intervention, such as directional atherectomy with distal embolic protection followed by balloon angioplasty. Local thrombolytic therapy may be required in cases of extensive thrombosis^134^. Other reported strategies include balloon angioplasty with thrombolysis, aspiration thrombectomy, and stent placement^134–136^. For suture-based closure devices (e.g., Perclose ProGlide), ischemia can result from dissection, thrombosis, or acute vessel occlusion due to suture-mediated closure. Management generally involves crossing the occlusion with a guidewire, followed by balloon angioplasty to restore flow^137^.

#### Deep vein thrombosis

Deep vein thrombosis (DVT) is a rare but clinically important complication following transfemoral catheterization, with reported incidence rates of approximately 0.05% in large series^138^. The underlying mechanism is primarily related to venous stasis and endothelial injury, most commonly driven by prolonged or excessive groin compression, access-site hematoma causing extrinsic venous compression, and postprocedural immobilization^138,139^. Case reports and observational data further suggest that prolonged manual compression may exacerbate components of Virchow’s triad, thereby increasing thrombosis risk despite intraprocedural anticoagulation^138,139^. Most cases can be managed conservatively with anticoagulation, typically low-molecular-weight heparin, with favorable outcomes; however, the true incidence may be underestimated due to asymptomatic presentations^138^.

## Complications common to all catheter-based procedures

### Stroke

Periprocedural stroke is a rare but devastating complication of PCI, with an incidence of approximately 0.1% to 0.5% for TRA^10,140,141^. Previous studies have shown that about half of these strokes are ischemic and the other half are hemorrhagic^142^. Predictors include both patient- and procedure-related factors. Patient-related factors include advanced age, female sex, prior stroke, diabetes, CKD, PAD, hypertension, left ventricular (LV) dysfunction, and presentation with ACS^140,142,143^. Procedural factors include use of intra-aortic balloon pump (IABP), PCI of bypass grafts, greater number of catheters used, rotational atherectomy, and the use of large-bore catheters^142–144^.

During the early adoption of TRA, stroke risk was a concern due to catheter manipulation potentially dislodging atheroma near the vertebral artery and the learning curve associated with more frequent catheter exchanges^142^. However, with increasing operator experience and improved technique, a large prospective study later reported that TRA was associated with a lower risk of stroke compared with TFA^140^. In contrast, a meta-analysis of randomized trials found no significant difference in stroke rates between the two approaches^10^.

Periprocedural ischemic strokes are most often embolic; brain MRI has been reported to show multiple acute infarctions in different vascular territories^145^. These lesions are typically characterized by scattered, small cortical or cortico-subcortical infarcts with bilateral or multiterritorial distribution, consistent with microembolic phenomena^146^. Neuroimaging studies demonstrate that the majority of PCI-related strokes exhibit an embolic pattern, most commonly involving the middle cerebral artery territory and frequently presenting as multiple infarcts^146^. Proposed mechanisms include dislodgement of atherosclerotic plaque or thrombus formation on catheters^147,148^. Strategies to reduce stroke risk include ensuring adequate systemic anticoagulation, minimizing catheter size and exchanges, and maintaining high operator proficiency with TRA.

### Radiation exposure

Although TRA is associated with clinical benefits, one concern is increased radiation exposure to both patients and operators. Earlier observational and randomized studies demonstrated that TRA was associated with longer fluoroscopy times compared with TFA^149^. However, a more accurate metric for radiation exposure is the dose-area product. A substudy from the RIVAL trial showed no significant difference in dose-area product between TRA and TFA overall, with the only subgroup showing higher dose-area product in TRA being low-case-volume centers^150^. In contrast, a large substudy of the MATRIX trial found higher radiation exposure with TRA compared with TFA, even though MATRIX was conducted exclusively at radial-experienced centers^151^. These differences may be explained by the smaller sample size and lower proportion of PCI cases in RIVAL, which reduces total fluoroscopy time and may limit the power to detect differences. A meta-analysis also demonstrated a small but statistically significant increase in radiation exposure with TRA compared with TFA^152^. These findings underscore the importance of strict radiation safety practices, particularly for operators.

### Acute kidney injury (AKI)

The reported incidence of AKI after PCI using TRA ranges from 5.5% to 21.5%^153–155^. One of the most important mechanisms of post-PCI AKI is contrast-induced AKI (CI-AKI). To prevent CI-AKI, the first step is risk assessment using validated tools such as the Mehran score^156^. The score incorporates non-modifiable risk factors, including advanced age, female sex, diabetes, history of heart failure (HF), and CKD, as well as modifiable risk factors including contrast volume, hypotension, anemia, preprocedural hyperglycemia, periprocedural hypovolemia, and use of an IABP^156^. Addressing modifiable risk factors before and during the procedure is critical.

Hydration remains the cornerstone of CI-AKI prevention. Standard protocols recommend isotonic saline at 1–1.5 mL/kg/hr for 3–12 h prior to PCI and 12–24 h postprocedure^157^. Individualized hydration guided by left ventricular end-diastolic pressure (LVEDP) has also been proposed and was shown to reduce the incidence of CI-AKI by 60% in a randomized trial^158^. This protocol involves a 3 mL/kg preprocedural bolus followed by LVEDP-guided infusion for 4 h postprocedure: 5 mL/kg/hr if LVEDP <13 mmHg, 3 mL/kg/hr if LVEDP 13–18 mmHg, and 1.5 mL/kg/hr if LVEDP >18 mmHg^158^. Notably, this trial excluded certain high-risk populations such as patients with ST-elevation myocardial infarction (STEMI) and decompensated HF.

Contrast volume correlates strongly with CI-AKI risk; every additional 100 mL of contrast increases the risk by 12–30%^159,160^. Contrast-sparing strategies include minimizing injection volume, using diluted contrast, coaxial or long catheters, echocardiography instead of ventriculography, smaller (5-Fr) catheters, biplane angiography, automated or contrast-reduction systems, staging complex PCI, and intravascular ultrasound (IVUS) guidance^161^. Finally, access-site complications leading to hemodynamic instability or bleeding can cause renal hypoperfusion, resulting in prerenal AKI and potentially acute tubular necrosis (ATN). Preventing access-site bleeding and hemodynamic compromise could potentially reduce the risk of AKI.

## Mechanism of radial vs femoral access in ACS

### AKI

The incidence of AKI after PCI is reported to range from 3% to 15%^160,162^. TRA has been associated with a reduced risk of AKI compared with TFA. A prespecified analysis of the large randomized MATRIX-AKI trial demonstrated a 13% relative risk reduction in AKI with TRA compared with TFA^155^. Subgroup analysis showed that this benefit was most pronounced in high-risk patients, particularly those with baseline renal dysfunction or a high Mehran CI-AKI risk score^155^. A large retrospective analysis also confirmed a greater reduction in AKI with TRA versus TFA among patients at higher baseline risk for kidney injury^163^.

Contrast volume is a well-established determinant of AKI risk^164^. Earlier studies noted that contrast use correlates with operator experience, especially in complex PCI^161^. Interestingly, the mechanism behind AKI reduction with TRA does not appear to be explained by lower contrast use, as contrast volume was not significantly different between TRA and TFA in the MATRIX-AKI analysis, which was conducted in radial-experience centers^155^. Multivariable regression suggested that the reduced AKI with TRA is largely driven by decreased major bleeding risk^155^. Patients at higher risk for bleeding, including those with low baseline glomerular filtration rate (GFR) and high CI-AKI risk scores, experienced the greatest renal protection with TRA^155^. Hemodynamically significant bleeding can cause renal hypoperfusion and ischemic injury. Another potential mechanism is reduced cholesterol embolization; TRA may minimize manipulation of catheters in the descending aorta near the renal arteries, lowering the risk of cholesterol microembolism compared with TFA, although this hypothesis was not directly assessed in MATRIX^155^.

### Mortality

Multiple large randomized trials have demonstrated that TRA is associated with lower all-cause mortality compared with TFA, particularly in high-risk groups such as patients with STEMI^3,4^. A recent meta-analysis of major randomized studies confirmed this survival benefit, showing a 24% relative risk reduction in mortality with TRA; notably, 95% of the pooled population had ACS, and 75% underwent PCI^5^. Several mechanisms may explain this mortality benefit. The most important factor is the reduction in major bleeding. TRA consistently lowers major bleeding risk compared with TFA, as demonstrated across randomized trials and confirmed by meta-analysis, which reported a 51% relative risk reduction^5^. Subgroup analyses have shown a mortality benefit of TRA across most patient categories, except in those without baseline anemia. Interestingly, TRA was associated with lower mortality in patients with significant anemia (hemoglobin <11 g/dL), whereas no significant difference was seen in patients without anemia^5^. Mediation analyses suggest that bleeding reduction partially, but not entirely, accounts for the survival advantage of TRA^5^.

Reduced need for blood transfusion may also contribute. Meta-analyses have shown that TRA is associated with fewer transfusions than TFA^5^. Prior studies have demonstrated that patients requiring transfusion after PCI have higher postprocedural mortality^165^. Lower vascular complication rates may further improve outcomes. TRA is associated with fewer vascular complications than TFA in meta-analyses^5^. Use of ultrasound-guided vascular access reduces puncture attempts, and randomized data show the consistent benefit of TRA in reducing vascular complication in setting of ultrasound-guided^66^. Finally, the lower incidence of AKI with TRA may contribute to its mortality benefit^155^. AKI is associated with morbidity and mortality and shares a bidirectional relationship with bleeding risk. Emergency procedures, which carry higher risks of vascular complications, AKI, and bleeding, may explain why survival benefit with TRA is most pronounced in high-risk populations such as STEMI.

## Current challenges

The impact of vascular complications on PCI outcomes remains significant, as these events can lead to major morbidity and mortality. Strategies to reduce complications should be considered across all procedural phases including preprocedural, intraprocedural, and postprocedural, but important gaps remain. In the preprocedural phase, the use of individualized risk stratification is still limited. Tools that integrate patient-specific clinical characteristics with vascular anatomy, when available, could improve access site selection and predict the risk of potential complications. The incorporation of large-scale data analytics and artificial intelligence may further refine prediction models and guide operators toward safer strategies tailored to each patient. During the procedure, several preventive measures are available but remain incompletely standardized and not universally adopted. Optimal intraprocedural anticoagulation strategies are also not fully defined. In the postprocedural phase, risk-adapted monitoring and follow-up strategies are not well established. Developing individualized postprocedural surveillance protocols may improve early recognition and management of complications, ultimately reducing adverse outcomes and preserving future vascular access options.

Alternative access sites have been explored to improve procedural outcomes and reduce access-site complications. Current guidelines support distal TRA, performed at the anatomical snuffbox, and transulnar access as alternative options to proximal TRA^12,166^. Randomized studies and meta-analyses have demonstrated that distal TRA reduces RAO and large hematomas compared with proximal TRA, albeit with longer cannulation time, more puncture attempts, and higher crossover rates^167,168^. Distal TRA may also improve patient comfort, facilitate hemostasis, and preserve the proximal radial artery^169^. A network meta-analysis of 47 randomized trials further confirmed that both distal TRA and transulnar access reduce bleeding and hematoma compared with TFA, with distal TRA associated with lower RAO but higher crossover^170^.

Transulnar access represents a reasonable alternative when radial access is not feasible, although it is technically more challenging due to its deeper location and proximity to the ulnar nerve. In a randomized trial (*n* = 902), the ulnar arm had high crossover rates; however, among successful cases, outcomes were noninferior to TRA for MACE and vascular complications at 60 days^171^. A meta-analysis of 5 randomized trials confirmed comparable efficacy with higher crossover and more puncture attempts, supporting its use in selected patients, ideally with ultrasound guidance^12,169^.

Transbrachial access has been increasingly utilized. In the early randomized ACCESS trial (*n* = 900), procedural success and procedural times were comparable across brachial, radial, and femoral approaches; however, brachial access was associated with higher rates of major access-site complications^172^. In a large contemporary BCIS registry, brachial access was predominantly used in higher-risk patients and was associated with increased adverse outcomes, bleeding, and vascular complications^173^. In contrast, a recent retrospective study of 300 patients with failed TRA demonstrated that transbrachial access had similar procedural success, procedural time, fluoroscopy time, and contrast use compared with TFA, with shorter access time and hospital stay, and lower rates of access-site complications, particularly hematoma^174^. Collectively, these findings suggest that, in experienced centers, transbrachial access may represent a feasible alternative following failed TRA. Further studies are needed to better define optimal access-site selection strategies and to determine the appropriate role of alternative vascular access approaches in contemporary PCI practice.

Artificial intelligence (AI) and machine learning (ML) have emerged as promising tools for risk stratification in interventional cardiology^175^. Contemporary AI-based clinical decision support systems integrate multiple preprocedural variables, including age, renal function, diabetes, and hemodynamic status, to provide individualized estimates of contrast-induced acute kidney injury and bleeding risk, and may also inform procedural recommendations such as contrast limits and access-site selection^176,177^. In a single-center retrospective study, implementation of an AI-based clinical decision support tool was associated with a reduction in postprocedural bleeding complications^176^. In a large multicenter PCI registry, ML models evaluated for periprocedural bleeding demonstrated only modest improvement in discrimination and reclassification compared with traditional NCDR-CathPCI risk scores^177^. Collectively, these findings suggest that integration of large-scale clinical data through AI/ML may enhance individualized risk stratification, support personalized access-site selection, and optimize procedural planning to reduce complications and improve outcomes. However, further studies are needed for model development, external validation, assessment of generalizability, and integration into clinical workflows.

## Conclusion

Although the TRA has become the standard access for PCI and has demonstrated significant reductions in bleeding, vascular complications, and mortality compared with the TFA, it still carries a risk of complications. TRA vascular complications, including radial artery perforation, RAS, and RAO, may negatively affect PCI outcomes and limit reaccess for repeat PCI or be reserved for conduit for CABG. In TFA, complications such as femoral pseudoaneurysm and retroperitoneal bleeding remain clinically important and can cause significant morbidity if not promptly recognized and treated. Further research is needed to reduce vascular complications through better patient selection and preprocedural planning, using tools to optimize procedural outcomes and appropriate postprocedural care and surveillance. Development and integration of individualized risk stratification tools, including the use of artificial intelligence to combine clinical and anatomical factors, may help guide access site selection, predict complications, and improve PCI outcomes.
